# Early Discharge after Delivery. A Study of Safety and Risk Factors

**DOI:** 10.1100/tsw.2003.108

**Published:** 2003-12-18

**Authors:** Deena R. Zimmerman, Gil Klinger, Paul Merlob

**Affiliations:** TEREM-Immediate Medical Services, Jerusalem, Macabi Health Services, Shaalvim, Israel

**Keywords:** maternal health, newborns, prenatal care, early discharge, hyperbilirubinemia, congenital heart disease, quality of care, Israel

## Abstract

The increased frequency of early discharge of newborns has led to questions of its safety. Most studies have looked at mortality and rehospitalization, not all missed diagnoses. The purpose of this study was to determine diagnoses in newborn infants that would have been missed if the infant had been discharged in <24 h. The design was a cohort study at Rabin Medical Center-Beilinson Campus (average monthly deliveries 1996 [250], 1997 [500]), a university-affiliated community hospital with all in-born term (≥37 weeks) infants born September through November 1996 and June 1997.

The main outcome measures were medical diagnoses (except trivial physical descriptions) noted at discharge (generally at ≥48 h) exam, not noted on admission exam (<24 h).

The results showed that 54 infants (5.1%) had diagnoses that were not detected before the infant was 24 h of age. The leading diagnosis was hyperbilirubinemia. Other potentially missed diagnoses included congenital heart disease (n = 10), morbidity of birth trauma (n = 9), metabolic disturbances (n = 2), hip dislocation (n = 1), suspected sepsis (n = 2), excessive weight loss (n = 2), polycythemia (n = 2), inguinal hernia (n = 1), and abducens paresis (n = 1).

It is concluded that diagnoses can be missed by discharging infants in 24 h or less. These diagnoses have the potential for adverse sequela. Even if early discharge is felt to be cost effective, parents should be counseled that it is not risk free. Better mechanisms should be put in place for assuring the safety of such infants.

## INTRODUCTION

The length of stay for newborns in the hospital nursery has become progressively shorter over the last 2 decades[1]. The American Academy of Pediatrics (AAP) currently defines an early discharge as occurring at ≤48 h of age and a very early discharge as occurring at ≤24 h of age. In the United States (U.S.), however, many insurance companies have mandated very early discharge as the norm[2].

While a number of studies have claimed to show the safety of early discharge, many of these studies are fraught with difficulties[3]. For example, many of the "early" group in these studies were hospitalized longer than the U.S. infants discharged later. In others, the groups were not randomly assigned or the eligibility criterion narrowly defined. Brumfield et al.[4] presented a study of early discharge in a large number of infants (n = 856) and mothers (n = 972), but while they were pleased with the safety and cost efficiency of their program, they demonstrated how few women (17%) and infants (15%) were actually eligible for early discharge, when careful guidelines were followed. A large study of the Washington State experience[5] demonstrated that newborns discharged early (<30 h after birth) were at increased risk of rehospitalization in the first month of life. Most other studies that focus on the difference in mortality and morbidity requiring rehospitalization lack the statistical power for this type of comparison[6,7] as 14,000 patients would be needed to detect a 25% increase in rehospitalizations at an underlying rehospitalization rate of 2%. Another problem with the focus on mortality and hospitalization-requiring morbidity is that other adverse outcomes are not considered. For example, one study failed to find an increased rate of hospitalization for feeding difficulties[8], but did not address adverse outcomes not leading to hospitalization such as early cessation of breastfeeding. While a current U.S. federal law has mandated that insurers pay for at least 48 h in the hospital, other countries do not have such a law and some such as Israel have been gradually shortening their maternity stays as well.

We report here a descriptive study in a setting where >48 h was still the norm for postnatal maternal/infant hospitalization. Infant diagnoses that were not found on the first day exam (and thus would have been missed had the newborns been discharged at 24 h or less), but found on the second day exam are reported. To the best of our knowledge this different approach to the problems of early discharge has not been previously used.

## METHODS

### Setting

Well baby and level II nursery of Rabin Medical Center, Beilinson Campus, Petach Tikva, Israel. In the first phase of the study, the delivery rate was 300/month. In November of 1996, the Beilinson Campus took over the patient load of the Golda Meir Campus leading to an increased delivery rate of 500/month and a more ethnically diverse population. In this nursery, all infants are examined on a daily basis by residents with the bedside assistance of the attendings.

### Subjects

The charts of all infants 37 weeks or greater born from September 1 to November 30, 1996 and admitted directly to the levels I and II nurseries were reviewed. To determine any differences after the merger, the charts of all full-term infants born June 1997 were reviewed as well.

### Data Abstracted

Length of stay (defined as 12 noon of day of discharge - hour of birth), method of delivery, parity, feeding method, and diagnoses other than simple physical diagnoses revealed during hospitalization. The definition of length of stay was chosen as all infants in this hospital were examined in the morning and the parents met with the attendings at 11 a.m. on the day of discharge. Thus all infants were discharged at approximately noon. Even if a child was physically on the hospital premises for longer, no further medical intervention would generally be done. A presumed diagnosis noted on admission that was further clarified during the hospitalization, such as a systolic murmur that was confirmed as a persistent ductus arteriosus or a prenatal diagnosis of hydronephrosis that was shown to be ureteropelvic junction obstruction, was not considered a missed diagnosis.

## RESULTS

In the 1996 study period, there were 715 live births, 595 (83%) of them meeting our study criteria. In June 1997, there were 527 live births, 464 (88%) meeting our study criteria. The mean length of stay for vaginal deliveries was 63 h (range 47–246, standard deviation 27) and for surgical deliveries 108 h (range 87–246, standard deviation 50). The demographics of the study subjects are shown in Table 1.

**TABLE 1**
**Demographic Variables of Study Population**

| Variable | Number | Percentage |
|---|---|---|
| Male | 555 | 52.4 |
| Vaginal deliveries | 820 | 77.4 |
| Firstborn | 361 | 34.1 |
| Breastfeeding (including partial) | 885 | 83.6 |

Of the 1,059 infants, 54 (5.1 %) had diagnoses that were not detected in <24 h of age that were subsequently diagnosed during their hospital stay. The leading diagnosis was hyperbilirubinemia. Overall, 30 infants were diagnosed with hyperbilirubinemia (>12 mg%) after the first day during the study period as depicted in Table 2; 15 received phototherapy. An additional 24 infants had notable findings discovered prior to discharge that were not discovered during the admission physical conducted in the first 24 h. The new diagnoses are detailed in Table 3. There was some overlap with one infant having both a VSD (Ventricular Septal Defect) and weight loss and two having both a fractured clavicle and jaundice.

## DISCUSSION

Our findings indicate that 5.1% of the study children would have had a missed diagnosis had they been sent home at <24 h of age. A study of repeat examinations in England showed 63/1,795 (3.5%) previously undetected abnormalities at hospital discharge[9]. These repeat examinations were in general performed later than those in our study so some problems may have become clinically obvious earlier thus leading to the lower percentage in that study in comparison with ours.

**TABLE 2**
**Hyberbilirubinemia After the First Day**

| No. | Gest Age | Gender | max bili | Cause | Photo |
|---|---|---|---|---|---|
| 1 | 39 | M | 15.0 | None | No |
| 2 | 38 | M | 15.7 | None | No |
| 3 | 40 | M | 15.7 | ABO | No |
| 4 | 39 | M | 15.9 | None | No |
| 5 | 39 | M | 15.0 | ABO | No |
| 6 | 38 | M | 15.0 | None | No |
| 7 | 39 | M | 16.9 | ABO | Yes |
| 8 | 39 | M | 18.0 | None | Yes |
| 9 | 37 | F | 14.5 | None | No |
| 10 | 41 | F | 18.0 | ABO | Yes |
| 11 | 41 | F | 16.7 | None | Yes |
| 12 | 37 | F | 14.5 | None | Yes |
| 13 | 38 | F | 14.3 | ABO | No |
| 14 | 40 | M | 14.3 | G6PD | No |
| 15 | 41 | M | 12.4 | None | No |
| 16 | 39 | M | 18.0 | Hemorrhage | Yes |
| 17 | 38 | F | 14.0 | ABO | No |
| 18 | 40 | F | 14.2 | ABO | No |
| 19 | 38 | F | 19.0 | ABO | Yes |
| 20 | 38 | F | 17.3 | ABO | Yes |
| 21 | 38 | F | 17.3 | None | Yes |
| 22 | 40 | F | 15.0 | ABO | Yes |
| 23 | 38 | F | 18.0 | Polycythemia | Yes |
| 24 | 37 | M | 14.3 | None | No |
| 25 | 38 | M | 15.6 | None | No |
| 26 | 38 | M | 17.2 | None | Yes |
| 27 | 40 | M | 16.7 | G6PD | No |
| 28 | 40 | F | 15.3 | ABO | Yes |
| 29 | 40 | F | 16.0 | ABO | Yes |
| 30 | 38 | F | 17.5 | None | Yes |

The significance of the missed diagnoses in our study could be debated, but in our opinion many could be important. The most common new diagnosis in our study was hyperbilirubinemia, a known concern of early discharge[10,11]. Among those infants with hyperbilirubinemia in our study, 15 received phototherapy. Most of the newborns who did not have ABO incompatibility began this phototherapy at more "liberal" levels of 17 mg%. None of those that received phototherapy, even those with risk factors for hyperbilirubinemia, had bilirubin levels high enough at 24 h to have justified a prolonged hospital stay. One might argue that they could have been discharged safely with home follow-up of the bilirubin. However, in one study, 10% of parents missed follow-up appointments despite signed contracts[12]. Home follow-up programs have missed such infants as well, often due to the lack of training of the healthcare personnel making the visit[13]. By the time parents notice the symptoms, it is often too late for intervention

**TABLE 3**
**Diagnoses Other than Hyperbilirubinemia Discovered After 24 h**

| Diagnosis | No. |
|---|---|
| VSD | 7 |
| Pulmonic stenosis | 1 |
| Tricuspid regurgitation | 1 |
| Bicuspid aortic valve/mild coarctation | 1 |
| Clavicular fracture | 8 |
| Hip dislocation | 1 |
| Hypocalcemia (<8 mg %) | 1 |
| Hypoglycemia (<44 mg %) | 1 |
| Fever | 2 |
| Adrenal hemorrhage | 1 |
| Weight loss >10% | 2 |
| Leukocytosis (55,000) | 1 |
| Inguinal hernia | 1 |
| Abducens paresis | 1 |
| Polycythemia | 2 |

and kernicterus can result[13,14]. Furthermore, the type of home follow-up programs (home visitation or health visitor) described in the literature supporting the safety of early discharge and home phototherapy are not available in many countries, such as Israel.

Ten infants would have had a pathologic cardiac diagnosis missed had they been discharged at <24 h. While the long-term course of the study infants is not known, at least two had a moderate VSD, which could have presented as congestive heart failure, if not noted. Alternatively, the parent may have noted feeding difficulties and stopped breastfeeding for the wrong reasons. Missing such a diagnosis in the hospital would not be a problem, if the next physical exam were within 24–48 h after hospital discharge. However, in Israel the next routine physical exam of the child in the context of well childcare provided by mother and child clinics is at 3 months of age. Although the AAP recommends that infants discharged at 48 h be examined within 2 days of discharge, research shows that even in the U.S. this is not being done[15].

Two breastfeeding infants were noted to have a greater than 10% weight loss (one 18%!). Breastfed babies that have gone on to have severe dehydration have attracted much attention in the popular as well as academic press[16,17,18,19]. Shortened hospital stays decrease the time available for proper breastfeeding education and support. While it has been mentioned that shortened hospital stays can actually improve breastfeeding by protecting new mothers from nonsupportive hospital practices[20], a better way to correct this would be to promote optimal breastfeeding practices such as those suggested by UNICEF and the WHO in the Baby Friendly Hospital Initiative[21].

Two infants had signs of potential sepsis — one had fever and one leukocytosis (55,000 per cubic mm). In the first infant, the blood culture grew staph epidermis that might be a pathogen in a newborn infant. The cultures of the second infant were negative.

Two infants had polycythemia, one requiring partial exchange transfusion and the other asymptomatic and untreated. Polycythemia and the attendant hyperviscosity can be a cause of devastating complications such as stroke or renal vein thrombosis and quite unlikely to have been noticed by parents untrained to look for this condition.

Additional diagnoses were discovered that may not have led to long-term medical difficulties, but could have been distressing to the parents. Eight infants had fractured clavicles, which were not noted on the first exam. This may have led to infants crying for unexplained reasons and anger on the part of the parents due to a missed diagnosis.

The missed developmental dysplasia of the hip is in concert with other studies of this phenomenon[22]. Once again, however, if the next physical exam of the infant is at 3 months of age, this could have important health consequences if missed.

Our study does not take into account diagnoses that could have been missed due to early metabolic screening[23]. Screening for PKU (phenylketonuria) <24 h after the first feeding leads to an increased need for repeat screening and infants who do not complete an adequate screen[24].

Even delaying discharge to beyond 48 h will not prevent all missed diagnoses and rehospitalization. During the 1996 study period, an additional three infants were discharged at >48 h and still needed readmission for phototherapy due to hyperbilirubinemia. There may also have been additional rehospitalizations of the study infants to other hospitals that we would have been unable to determine by our study design. Discharge of the study patients after the first physical exam, however, would have increased the possibility of missed diagnoses. Therefore discharge at <48 h should not be presented to parent as a risk free procedure.

## CONCLUSIONS

Early discharge can lead to missed diagnoses and thus should not be presented as a risk free procedure. The potential health disadvantages of early discharge need to be carefully balanced against the economic advantages. Our findings add information as to what diagnoses may be missed by early discharge that would not appear as rehospitalization statistics. Our personal feelings are that very early discharge (<24 h) should be completely avoided and only early discharge of 48 h may be considered for a full term appropriate for gestational age infant. A home visit at 48 h postdischarge by a nurse with newborn nursery experience should be a sine-qua-non for early discharge. The ultimate discharge decision should be based on individual clinical considerations combined with informed parental choice, not third-party considerations.

## RERERENCES

1. Kessel, W., Kiely, M., Nora, A.H., and Sumaya, C.V. (1995) Early discharge, in the end, it is judgment. *Pediatrics* **96,** 739–742.
2. Annas, G.J. (1995) Women and children first. *N. Engl. J. Med.* **33**, 1647–1651.
3. Beck, C.T. (1991) Early postpartum discharge programs in the United States: a literature review and critique. *Women Health* **17**, 125–137.
4. Brumfield, C.G., Nelson, K.G., Stotser, D., Yarbaugh, D., Patterson, P., and Sprayberry, N.K. (1996) 24-hour mother-infant discharge with a follow up home health visit results in a selected medicaid population. *Obstet. Gynecol.* **88**, 544–548.
5. Liu, L.L., Clemens, C.J., Shay, D.K., Davis, R.L., and Novack, A.H. (1997) The safety of newborn early discharge: the Washington State experience. *JAMA* **278**, 293–298.
6. Braverman, P., Egerter, S., Pearl, M., Marchi, K., and Miller, C. (1995) Early discharge of newborns and mothers: a critical review of the literature. *Pediatrics* **96**, 716–726.
7. Braveman, P., Kessel, W., Egerter, S., and Richmond, J. (1997) Early discharge and evidence based practice: good science and good judgment. *JAMA* **278,** 334–336.
8. Edmonson, M.B., Stoddard, J.J., and Owens, L.M. (1997) Hospital readmission with feeding related problems after early postpartum discharge of normal newborns. *JAMA* **278**, 299–303.
9. Moss, G.C., Cartlidge, P.H.T., Speidel, B.D., and Chambers, T.L. (1992) Routine examination in the neonatal period. *BMJ* **302**, 878–879.
10. Catz, C., Hanson, J., Simpson, L., and Yaffe, S. (1995) Summary of workshop: early discharge and neonatal hyperbilirubinemia. *Pediatrics* **96**, 743–745.

11. Seidmaan, D.S., Stevenson, D.K., Ergaz, Z., and Gale, R. (1995) Hospital readmission due to neonatal hyperbilirubinemia. *Pediatrics* **96**, 727–729.
12 Conrad, P.D., Wilkening, R.B., and Rosenberg, A.A. (1989) Safety of newborn discharge in less that 36 hours in an indigent population. *Am. J. Dis. Child.* **143**, 98–101.
13. MacDonald, M.F. (1995) Hidden risks: early discharge and bilirubin toxicity due to glucose 6 phosphate dehydrogenase deficiency. *Pediatrics* **96**, 734–745.
14. Maisels, M.J. and Newman, T.B. (1996) Kernicterus in otherwise healthy, breast-fed term newborns. *Pediatrics* **96**, 730–733.
15. Maisels, M.J. and Kring, D. (1997) Early discharge from the newborn nursery-effect on scheduling of follow-up visits by pediatrician. *Pediatrics* **100**, 72–74.
16. Whitlow, J. (1994) Insurer limits on maternity stay reshaping care. *The Sunday Star-Ledger* June 5.
17. Lawrence, R. (1995) Early discharge alert. *Pediatrics* **96**, 966–967.
18. Pascale, J.A., Brittan, L., Lenfestey, C.C, and Jarrett-Pulliam, C. (1996) Breastfeeding, dehydration and shorter maternity stays. *Neonatal Netw.* **15**, 37–43.
19. Cooper, W.O., Atherton, H.D., Kahan, M., and Kotaggal, U.R. (1995) Increased incidence of severe breastfeeding malnutrition and hypernatremia in a metropolitan area. *Pediatrics* **96**, 957–960.
20. Zimmerman, D.R. and Bernstein, W.R. (1996) Standing feeding orders in a well baby nursery: “Water, water everywhere.” *J. Hum. Lact.* **12**, 189–192.
21. World Health Organization (1989) Promoting a Supporting Breast-Feeding: The Special Role of Maternity Services. A Joint WHO/UNICEF Statement. Geneva, Switzerland.
22. Cartlidge, P.H.T. (1992) Routine discharge examination of babies: is it necessary? *Arch. Dis. Child.* **67**, 1423–1429.
23. Sinai, L.N., Kim, S.C., Casey, R., and Pinto-Martin, J.A. (1995) Phenylketonuria screening: effect of early newborn discharge. *Pediatrics* **96**, 605–608.
24. Bernstein, W.R., Krauser, D.G., and Rhoads, F.A. (1996) Effects of early newborn discharge on neonatal screening. *Arch. Pediatr. Adolesc. Med.* **150(Suppl.),** 86.

## BIOSKETCHES

**Deena R. Zimmerman**, **M.D., M.P.H.,** is a specialist in pediatrics with clinical work at the TEREM-Immediate Medical Services in Jerusalem and Macabi Health Services in Shaalvim. At the same time, Dr. Zimmerman does research work at the National Institute of Child Health and Human Development, Saban Children’s Medical Center, Soroka University Medical Center, Beer-Sheva, Israel. E-mail: yoatzothalacha@nishmat.net

**Gil Klinger, M.D.,** is a Fellow in Neonatology at the Department of Neonatology, Schneider Children’s Medical Center, Petach Tikva, Israel. E-mail: gilkl@post.tau.ac.il

**Paul Merlob, M.D.,** is Professor of Pediatrics at the Sackler School of Medicine, Tel Aviv University, Tel Aviv and Director of the Department of Neonatology, Schneider Children’s Medical Center of Israel, Petach Tikva, Israel. E-mail: pmerlob@clalit.org.il
